# miRNAs as potential biomarkers for early detection of prediabetes among obesity: a systematic review

**DOI:** 10.1042/BSR20253283

**Published:** 2025-12-16

**Authors:** Brenda Wen Keng-Lim, Kelvin Yee Chen-Ng, Jia Yee-Lee, Ching Hsein-Chen, Wan Ling-Chang, Rhun Yian-Koh, Anil Philip-Kunnath, Soi Moi-Chye

**Affiliations:** 1 School of Health Science, IMU University, No. 126, Jalan Jalil Perkasa 19, Bukit Jalil, Kuala Lumpur, 57000, Malaysia; 2 Department of Microbiology, Immunology and Biopharmaceuticals, College of Life Sciences, A25-303 Room, Life Sciences Hall, No. 300, Syuefu Road, National Chiayi University, Taiwan; 3 Department of Anaesthesiology, Chang Gung Memorial Hospital at Chiayi, No. 8, West Section of Jiapu Road, Puzi City 613016, Chiayi County, Taiwan; 4 School of Health Science, Division of Biomedical Science and Biotechnology, IMU University, No. 126, Jalan Jalil Perkasa 19, Bukit Jalil, Kuala Lumpur, 57000, Malaysia

**Keywords:** diagnostic accuracy, impaired glucose tolerance, impaired fasting glucose, microRNA, miRNA, metaanalysis., obesity, prediabetes, ROC, sensitivity, specificity

## Abstract

**Background:** Prediabetes in individuals with obesity is a high-risk state for progression to type 2 diabetes mellitus (T2DM). Circulating microRNAs (miRNAs) have emerged as promising minimally invasive biomarkers for early detection. However, their diagnostic performance and consistency across studies remain unclear. **Objectives:** To evaluate circulating miRNAs as potential biomarkers for prediabetes in obese populations. **Methods:** A systematic search of PubMed, MEDLINE, Scopus, and EBSCOhost was conducted (September 2012–September 2025) without language restrictions. Eligible studies included observational, clinical, and translational research assessing circulating miRNAs in plasma, serum, whole blood, or exosomes using qRT-PCR, ddPCR, microarray, or next-generation sequencing. Two reviewers independently screened studies and extracted data using a piloted form. Extracted information was synthesized qualitatively; diagnostic performance measures and reported miRNAs were tabulated. **Results:** Nine circulating microRNAs (miR-27, miR-30a, miR-34a, miR-93, miR-122, miR-126, miR-146a, miR-192, and miR-193b) were consistently dysregulated in obese individuals with prediabetes across thirteen included human studies. These miRNAs were linked to key pathogenic mechanisms including chronic inflammation, insulin resistance, β-cell dysfunction, and altered adipokine signaling. Notably, inflammation-associated miRNAs (miR-27, miR-34a, miR-146a) reflected the transition from metabolically healthy to unhealthy obesity, while β-cell–related miRNAs (miR-30a, miR-126) indicated early impairment of insulin secretion. Among detection platforms, qRT-PCR remained the most sensitive and specific method for miRNA quantification, whereas microarray and next-generation sequencing provided broader profiling capability but with higher cost and complexity. **Conclusions:** Circulating miRNAs demonstrate promise as diagnostic biomarkers for prediabetes in obesity. However, these findings are limited by methodological variability. Thus, large-scale and standardized studies are required to validate their clinical utility.

## Introduction

Diabetes mellitus (DM) is a chronic metabolic disorder characterized by persistent hyperglycemia. Chronic hyperglycemia not only affects the secretion and action of insulin but also leads to various tissue and organ dysfunctions [1]. To date, 96% of global diabetes cases are diagnosed as type 2 diabetes mellitus (T2DM) [2]. T2DM is characterized by defective insulin secretion by pancreatic β-cells and an impaired tissue response to insulin, leading to hyperglycemia [3]. The development of T2DM takes years and is often asymptomatic due to compensation mechanisms, making early diagnosis a challenge [4]. A transition stage that occurs before T2DM is known as prediabetes, a condition in which the blood glucose levels of patients are higher than normal, but below the diagnostic level of T2DM [5]. A major risk factor for the development of prediabetes is obesity. A recent study described a close relationship between obesity and T2DM, in which they share a common pathophysiological mechanism [6]. Obesity contributes to the pathogenesis of prediabetes due to the overaccumulation of lipids in adipose tissues, triggering protective mechanisms that lead to insulin resistance [7]. In obese individuals, glycerol, nonesterified fatty acids, and pro-inflammatory cytokines released by adipose tissue are elevated, which play a role in the development of insulin resistance [8]. Furthermore, obesity can lead to pancreatic islet cell stress via multiple mechanisms. A study shows that peripheral insulin resistance leading to chronic overproduction of protein causes endoplasmic reticulum (ER) stress that will result in pancreatic β-cell stress [9]. Since persistent β-cell stress ultimately leads to β-cell failure, it is crucial to detect β-cell stress before irreversible β-cell dysfunction and degeneration [4]. The complex pathogenesis of prediabetes and insulin resistance from various mechanisms are keys for the investigation of potential biomarkers for early detection. Currently, the management of prediabetes focuses on lifestyle changes. However, delayed diagnosis often happens as metabolic imbalances that lead to impaired insulin sensitivity at the prediabetic stage usually occur years before significant changes in glucose level can be detected [10]. Therefore, early detection of prediabetes is crucial, tracking it starting from obese individuals for early intervention in the prevention of prediabetes and T2DM.

**Figure 1:**
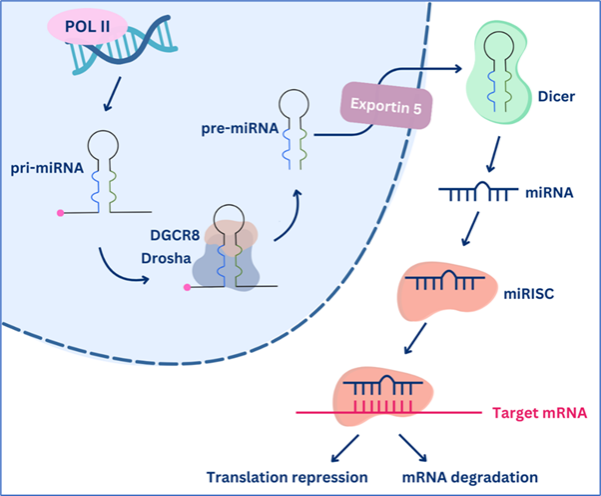
Biogenesis of miRNA. In the nucleus, pri-miRNA will be cleaved by DGCR8 and Drosha enzyme into pre-miRNA, following its export to the cytoplasm. In the cytoplasm, the Dicer enzyme processes the pre-miRNA into a single-stranded mature miRNA. To exert its function, miRNA will be integrated into an RNA-induced silencing complex (RISC).

A potential diagnostic biomarker that brings attention to researchers in the early detection of diabetes is microRNA (miRNA) [11]. miRNAs are a class of small, single-stranded, noncoding RNA molecules with 18–25 nucleotides in length that play important roles in post-transcriptional gene regulation [12]. The biogenesis of miRNA starts in the nucleus, where a primary miRNA (pri-miRNA) transcript is generated by RNA polymerase II, followed by cleavage into a pre-miRNA by a complex consisting of Drosha enzyme and DiGeorge Syndrome Critical Region 8 (DGCR8). The pre-miRNA is then transported into the cytoplasm via exportin 5, followed by subsequent processing by the Dicer enzyme to produce a single-stranded mature miRNA. Finally, mature miRNA is integrated into an RNA-induced silencing (RISC) complex to activate its function in gene regulation (Figure 1). miRNAs exert their functions by binding to the 3′-untranslated region of the target mRNA, leading to repressed translation or mRNA degradation [13,14]. An increasing number of studies have demonstrated the involvement of miRNAs in the development of a wide range of diseases in addition to diabetes, including cancer, neurodegenerative diseases, and infectious diseases [15]. miRNAs' regulatory role, disease association, tissue specificity, and stability in body fluids support their potential as biomarkers for the early detection of diseases [16].

While prior reviews have summarized the role of circulating miRNAs in diabetes broadly, they largely emphasized established type 2 diabetes and its complications. However, far less attention has been devoted to the prediabetic stage, particularly within the context of obesity, where early detection may have the greatest preventive impact. Our review addresses this gap by systematically examining obesity-associated miRNAs as candidate biomarkers for prediabetes, with a focus on mechanistic insights, diagnostic performance, and translational readiness.

In this review, we will first discuss the current diagnostic methods for prediabetes and address the challenges in identifying prediabetes among obese individuals. Then describe the dysregulation of specific miRNAs in prediabetes and obesity while exploring the molecular mechanisms involved. Furthermore, we will discuss the potential and challenges of miRNAs as early diagnostic biomarkers, evaluate the diagnostic performance of individual miRNAs and multimiRNA panels for detecting prediabetes among obese individuals. Then, we also discuss how sex, age, ethnicity, and metabolic phenotype affect accuracy. Finally, we will evaluate current miRNA detection methods, contextualize our findings against prior systematic reviews and clinical standards, and provide suggestions for future research.

## Current diagnostic approaches and challenges in identifying prediabetes among obese individuals

### Current diagnostic methods for prediabetes

The American Diabetes Association (ADA) and the US Preventive Services Task Force (USPSTF) have updated their guidelines for prediabetes screening, lowering the recommended screening age to 35 years and advising repeated screening every three years for asymptomatic individuals. For overweight or obese adults, screening is recommended at any age [17,18].

Current conventional diagnostic methods for prediabetes include measuring glycated hemoglobin A1c (HbA1c), fasting plasma glucose (FPG), and oral glucose tolerance test (OGTT). These diagnostic measures are clinically practiced due to high sensitivity and low specificity and do not differ by race or ethnicity [19]. The diagnostic criteria for prediabetes stated below are mainly based on ADA and WHO guidelines [20]. First, HbA1c levels of 5.7% to 6.4% are considered prediabetes [21]. HbA1c is known as the standardized measurement for prediabetes due to its simplicity and reliability. HbA1c reflects average glycemia throughout two to three months. However, its performance is still under discussion as its levels can be affected by medical conditions such as anemia and kidney dysfunction [20]. Another study agrees that measuring HbA1c alone can lead to under or overdiagnosis of prediabetes; hence, it must be coupled with other tests [22].

Next, both FPG and OGTT are glucose-based tests in the screening of prediabetes. The measurement of FPG reflects current levels of glucose. Prediabetes is diagnosed when the level lies between 100 and 125 mg/dl (5.6–6.9 mmol/l), which implies impaired fasting glucose [17]. However, there is a discrepancy between ADA and WHO guidelines in the cut-off value of FPG. The cut-off value of WHO for impaired fasting glucose is 110 mg/dl (6.1 mmol/l), which is lower than ADA criteria [23]. The limitation of FPG lies in its requirement to fast for at least 8 hours before the test. Inability to do so will lead to inaccuracy of test results. Moreover, FPG levels may be affected by medications such as glucocorticoids, anti-hypertensive drugs, and thyroid medications [20,24,25], whereas OGTT is a test to evaluate glucose tolerance as it assesses the body’s ability to metabolize and store glucose. The procedure of OGTT includes oral ingestion of 75 g glucose followed by measurement of blood glucose level after 2 h [17,26]. The diagnostic criteria for prediabetes are OGTT between 140 and 199 mg/dl (7.8–11 mmol/l). Although OGTT is known as the gold standard test according to WHO, most prediabetic individuals typically have glucose levels lower than the current diagnostic threshold of OGTT, leading to misdiagnosis [20,27].

Comparing these diagnostic tests for prediabetes, HbA1c has better accuracy than FPG as it reflects glycemic status over a few months considering prediabetes individuals might not have significant glucose spikes due to compensatory mechanisms. Moreover, HbA1c advantages include convenience as it does not require fasting before the test, better preanalytical stability, and less disturbance during stress or diet changes. Unfortunately, high cost and limited availability restricted its usage [20]. As to compare HbA1c with OGTT, a study on Thai patients suggested that OGTT has higher performance compared with HbA1c in screening high-risk patients, validating its claim as the gold standard test for prediabetes diagnosis [28].

### Challenges in identifying prediabetes among obese individuals

Identifying prediabetes among obese individuals poses significant challenges due to the complexity contributed by various factors. First, the risk of prediabetes is heterogeneous among obese individuals. Although many studies have consistently shown that a high BMI is associated with an increased risk of diabetes and metabolic syndrome, the relationship between BMI and metabolic disease is not always straightforward. A study discussing the concept of ‘metabolically healthy obese’ (MHO) shows that not all obese individuals may exhibit metabolic issues despite their BMI [29]. However, a longitudinal study conducted on middle-aged men observed that MHO men were at increased risk for diabetes too [30]. The factors that discriminate prediabetes risk within obese individuals are not well-characterized, making it difficult to predict who will progress to prediabetes. Furthermore, prediabetes is often asymptomatic, unlike T2DM, which will show symptoms like increased urination and unexplained weight loss [31].

Second, the diagnostic criteria for prediabetes are not standardized universally, particularly for impaired fasting glucose. WHO and ADA hold different cut-off values for the FPG test [17,23]. In addition, different tests may cause discordance in diagnosis depending on the criteria used, and HbA1c potentially identifies a higher prevalence of prediabetes compared with FPG. The lack of agreement on diagnostic criteria makes it challenging to identify prediabetes consistently [32]. Moreover, hyperinsulinemia in obese individuals may go undetected as it is not included in routine tests [33]. Third, the development of prediabetes in obese individuals is associated with insulin resistance and ectopic visceral fat deposition. A study suggested that excess visceral fat and insulin resistance may be more important mediators of prediabetes risk than BMI [34]. Since obese individuals with similar BMI can exhibit different metabolic profiles, measuring visceral fat instead of just BMI or total body fat may be needed for better prediabetes risk assessment in obese individuals. Finally, prediabetes screening rates remain suboptimal despite the high prevalence of obesity and prediabetes. A 3 year prospective study found out that at least one in four obese or overweight U.S. adults did not receive glucose testing although they are eligible for screening [35]. Therefore, increased screening efforts and personalized risk assessments are needed to detect prediabetes earlier in this high-risk group.

Early detection is of utmost importance as prediabetes can be reversed by lifestyle intervention. An outcome study conducted by the Centers for Disease Control and Prevention (CDC) has shown that prediabetes individuals who lost 5–7% of weight reduced their risk of developing T2DM by 58% [36]. To overcome the challenges and limitations of current diagnostic methods for prediabetes, it is necessary to search for a multifaceted approach that combines biomarkers with comprehensive risk assessment to achieve early detection. As mentioned, detecting β-cell stress before its loss of cell mass is crucial in the early detection of prediabetes. Unfortunately, there are no noninvasive biomarkers for early detection of β-cell dysfunction that are clinically validated [4].

## Search methodology for clinical studies (PRISMA 2020 compliant)

This systemic review was conducted in accordance with the PRISMA 2020 guidelines. No protocol was registered for this review.

We included observational human studies, clinical trials, and experimental translational studies that evaluated the expression levels of circulating miRNAs in plasma, serum, whole blood, or exosomes, using platforms such as real-time quantitative polymerase chain reaction (qRT-PCR), droplet digital polymerase chain reaction (ddPCR), microarray, or next-generation sequencing. Eligible populations include individuals with overweight or obesity, or mixed populations with extractable obese subgroup data, clinically classified as normoglycemic, prediabetic, or type 2 diabetic according to American Diabetes Association (ADA) or World Health Organizations (WHO) criteria. We excluded nonhuman studies, tissue-only studies, studies without prediabetes outcomes, noncirculating miRNAs, reviews, editorials, conference abstracts, and studies with insufficient extractable data.

Literature searches were conducted in MEDLINE (Ovid), PubMed, Scopus, and EBSCOhost Research Databases between September 2012 and September 2025. No language restrictions were applied, and translations were attempted where feasible.

The search strategy was developed with a medical librarian and peer-reviewed according to the PRESS guidelines, using the following keywords: miRNA, microRNA, circulating microRNA, prediabetes, prediabetes, impaired glucose tolerance, impaired fasting glucose, obesity, obese, and overweight. A combination of Medical Subject Heading (MeSH) terms and free-text terms was applied in the PubMed search.

**Figure 2:**
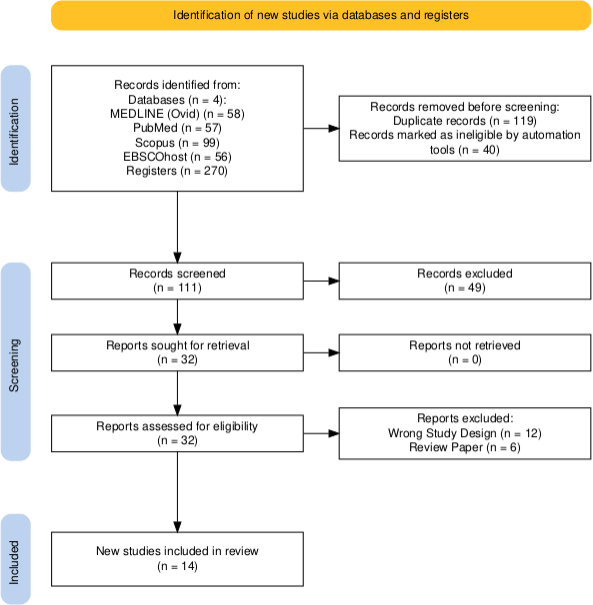
PRISMA flow diagram for literature search.

The retrieved articles were manually screened by two reviewers independently for relevant and potentially eligible studies. Preliminary selection of articles was done by screening title and abstract, followed by full-text review to evaluate relevance and eligibility for final inclusion or exclusion. Disagreements were resolved by consensus or by consulting a third reviewer, and the study selection process was documented using the PRISMA 2020 flow diagram (Figure 2).

Data extractions were performed by two reviewers using a piloted form. Extracted information was saved in the Excel computer software (Microsoft ® Office Excel) for subsequent classification and analysis. For studies reporting multiple miRNAs, each was treated as a separate index test, while panels of correlated miRNAs were extracted as composite tests.

The extracted data underwent qualitative synthesis. Information such as study characteristics, populations, and index tests were summarized narratively, while diagnostic performance outcomes were expressed numerically. The diagnostic accuracy metrics and the frequency of reported miRNAs were organized in tables to evaluate consistency across studies. Notably, meta-analysis was not performed due to the heterogeneity in study design, population characteristics, and laboratory methods.

## Results

### Literature research results

#### Study characteristics

A total of 13 studies were included (Table 1), enrolling approximately 2595 participants. Across the studies, there were 1,719 females and 876 males, though the balance varied considerably by cohort. Three studies recruited only women [38,40,47], one study included only male [41], while others reported more equal distributions of gender. Larger cohorts tended to be female-dominated, 346 men and 924 women [44]. Sample sizes varied considerably, from small, targeted cohorts such as obese women in Germany [47] (*n* ≈ 40–50) to large, multiethnic cohorts in the U.S. Diabetes Prevention Program (*n* > 1000) [49]. Pediatric participants were studied in Austria [45], while older adults were examined in Poland [48].

**Table 1:** Characteristics of included studies

Author (Year, Country)	Design and setting	Population (*n*) and demographics	Prediabetes definition	Index miRNA(s) / Panel	Specimen and platform	Normalization	Threshold	AUC (95% CI)	Notes
Kornfeld et al., 2013 (Germany) [37]	Experimental (animal and human translational); Human study: liver biopsies from lean and obese individuals	Lean and obese human subjects; *n* NR	NR	miR-802	Human liver biopsy; qRT-PCR	U6	NR	NR	-
Mehanna et al., 2015 (Egypt) [38]	Cross-sectional case-control study of Egyptian women (Metabolic syndrome vs. healthy controls); Outpatient Clinic Ismailia General Hospital and Suez Canal University Hospital	Egyptian women, metabolic syndrome vs. healthy controls; *n* NR	NR	miRNA-146a rs2910164 SNP	Whole blood; PCR–RFLP	NR (DNA genotyping study)	NR	NR	-
Nunez Lopez et al., 2016 (United States) [39]	Cross-sectional comparison of circulating cytokine and microRNA levels across groups; participants from ORIGINS trial (USA)	Healthy controls, prediabetes, T2DM; stratified lean vs. obese; *n* NR	ADA criteria: FPG 100–125 mg/dl, HbA1c 5.7–6.4%	miR-21, miR-24.1, miR-27a, miR-34a, miR-126, miR-146a, miR-148a, miR-152, miR-223, miR-25, miR-93, miR-150	Plasma; qRT–PCR (TaqMan)	Spike-in: cel-miR-39; endogenous control: miR-191, miR-423–3p, and miR-451	> 35 excluded and set to NA	NR	-
Manning et al., 2019 (New Zealand) [40]	Prospective interventional study: obese individuals underwent 8 week very-low-calorie diet (800 kcal/day)	Obese individuals recruited from University Duisburg-Essen outpatient clinic	ADA criteria: FPG 100–125 mg/dl, 2h-OGTT 140–199 mg/dl, HbA1c 5.7–6.4%	miR-30a, miR-499–5P, miR-126–3P, miR-566, miR-147a, miR-433–3p, miR-4516, miR-215–5p, miR-630, miR-568, miR-193a, miR-194–5p, miR-642–5p	Plasma; qRT–PCR (TaqMan)	Spike-in: cel-miR-39–3p	2−∆∆CT method, ≥ 35 eliminated	NR	-
Ghai et al., 2019 (Finland) [41]	Prospective cohort (METSIM study, Kuopio Finland)	Prediabetic men (isolated IFG) aged 45–70, followed 5 years for progression to T2DM	Isolated IFG: FPG 5.6–6.9 mmol/l	miR-122–5p, miR-127–5p, miR-136–5p, miR-192–5p, miR-210–3p, miR-4532–5p, miR-483–5p, miR-7641–3p	Plasma; qRT–PCR (TaqMan)	Normalizers: miR‐21–5 p and miR‐16–5 p	−ΔΔCT values, > 35 not considered	miR-122–5p: 0.66 (All), 0.60 (Normal BMI), 0.67 (Obese BMI); miR-127–5p, miR-136–5p, miR-210–3p, miR-4532–5p, miR-483–5p, miR-7641–3p: All 0.64 (Normal BMI 0.58, Obese BMI 0.81)	Internal validation
Corona-Meraz et al., 2018 (Mexico) [42]	Cross-sectional observational study, age groups compared	Adults stratified as young vs. senior, by insulin resistance status	NR	miR-33a, miR-33b	Serum; qRT-PCR	Endogenous reference gene: hsa-miR-320a	NR	miR-33a: Senior 0.804, Young 0.571 (95% CI: 0.655–0.952); miR-33b: Senior 0.798, Young 0.630 (95% CI: 0.640–0.955)	-
García-Jacobo et al., 2019 (Mexico) [43]	Cross-sectional observational study	Normal glucose tolerance, prediabetes, screen-detected diabetes; *n* NR	ADA 2017 criteria	miR-146a, miR-34a, miR-375	Serum; qRT-PCR (TaqMan)	Global mean	2−ΔCT method	NR	-
Weale et al., 2020 (South Africa) [44]	Cross-sectional observational study; pediatric outpatient clinic	Children with normal, prediabetes, screen-detected diabetes	FPG ≥ 100 mg/dl	miR-30a-5p, miR-182–5p	Serum; qRT-PCR (TaqMan)	cel-miR-39	2−∆∆CT method	miR-30a-5p: 0.69; miR-182–5p: 0.74	-
Lischka et al., 2021 (Austria) [45]	Prospective observational pediatric cohort	Children with severe obesity	International guidelines: FPG 100–125 mg/dl, 2 h OGTT 140–199, HbA1c 5.7–6.4%	miR-34a, miR-122, miR-192	Serum; QuantStudio 7 Flex qRT-PCR	miR-16-5p	2−ΔCt method	NR	-
Sardu et al., 2021 (Italy) [46]	Prospective, placebo-controlled interventional study	Obese patients with prediabetes undergoing metformin vs. placebo	IFG ≥ 5.6 mmol/l (prediabetes definition specific to IFG vs. NGT)	miR-195, miR-27	Serum; qRT-PCR	Mean of miR-16, 24, 25, 26	−ΔCT values	NR	-
Kovac et al., 2023 (Germany) [47]	Cross-sectional translational study	Obese women with IFG vs. NGT	FPG 5.6–6.9 mmol/l or HbA1c 5.7–6.4%	miR-652–3p, miR-877–5p, miR-93–5p, miR-130a-3p, miR-152–3p, let-7i-5p	Whole blood; qRT-PCR	cel-miR-39–3p	2−ΔΔCt method	NR	Validation: Melting curve analysis
Włodarski et al., 2024 (Poland) [48]	Cross-sectional hospital-based study	Elderly patients with carbohydrate metabolism disorders vs. controls	Fasting glucose: 95–125 mg/dl; 2 h OGTT: 140–199 mg/dL	miR-196a	Serum; qRT-PCR	Global mean Ct	−ΔCT values	0.5017 (*P*=0.977)	Validation: Negative control; replication: without chronic metabolic disease
Flowers et al., 2024 (United States) [49]	Longitudinal biomarker association study within RCT (DPP)	Participants from DPP trial; followed baseline, year 1, year 2	FPG 5.6–6.9 mmol/l or HbA1c 5.7–6.4%	miR-126, miR-15a, miR-192, miR-197, miR-23a, miR-27a, miR-320a	Plasma; qRT-PCR	hsa-miR-103a-3p and hsa-miR-16–5p	NR	NR	-

The studies selected included diverse geographical regions, including Germany, Egypt, New Zealand, Finland, Mexico, South Africa, Austria, Italy, Poland, and the United States. European populations (Italy, Germany, Poland, Finland, Vienna) were most frequently represented, patients from Germany, France, and Austria, the TÜF cohort of white German women, a Naples (Italy) cohort, pediatric participants from Austria, and additional studies from Germany, Finland, and Poland. Whereas studies from Mexico and South Africa contributed important insights into non-European groups. Notably, only one study specifically investigated an Egyptian population, highlighting regional genetic polymorphisms (miR-146a rs2910164) linked to metabolic risk [38]. The inclusion of diverse populations strengthens generalizability, though underrepresentation of Asian cohorts remains a limitation.

Prediabetes definitions were predominantly based on American Diabetes Association (ADA) criteria, including impaired fasting glucose (100–125 mg/dl), impaired glucose tolerance (2 h OGTT 140–199 mg/dl), or HbA1c 5.7–6.4%. However, not all studies reported explicit diagnostic thresholds, with two omitting cut-offs entirely [37,42].

The circulating miRNAs investigated included both individual candidates (miR-34a, miR-126, miR-146a, miR-192, miR-30a, miR-93, miR-122) and broader multimarker panels. These miRNAs were functionally linked to insulin signaling, β-cell dysfunction, adipose tissue inflammation, and oxidative stress. The mechanistic insights will be discussed further in the following section.

Specimen sources were mainly serum or plasma, profiled on qRT-PCR platforms. Reporting of preanalytical handling was inconsistent. Some studies specified overnight fasting before blood draw [42,45], and one incorporated hemolysis control using the miR-451/miR-23a ratio [47], but the majority did not describe preanalytics. Normalization strategies were heterogeneous, ranging from endogenous controls (U6 and miR-16) to exogenous spike-ins (cel-miR-39) or global mean scaling. CT thresholds were variably applied, mostly using the 2−∆∆CT method, with several studies excluding signals above 35 cycles as nonexpressed.

Diagnostic performance was modest to moderate overall. Reported AUC values ranged between 0.60 and 0.81. The strongest performance was observed in obese subgroups of the Diabetes Prevention Program, where miR-127–5p, miR-136–5p, miR-210–3p, and miR-483–5p each achieved an AUC of 0.81 [41]. In contrast, miR-196a showed no discriminatory ability (AUC = 0.50) in an elderly Polish cohort [48]. Sensitivity and specificity estimates were rarely reported, and thresholds lacked standardization across studies. External replication was largely absent, although limited internal validation was attempted in selected cohorts [41,48].

### MicroRNAs identified for prediabetes

#### Mechanisms and specificity considerations

Nine microRNAs that are related to both obesity and prediabetes that are mentioned two times or more in the papers were identified (Table 2). Only human studies that showed significant dysregulation of miRNAs in the blood samples of obese prediabetes individuals were included. miRNAs play a significant role in the pathogenesis of obesity and prediabetes by influencing various metabolic pathways and cellular functions. The role of miRNA in the pathogenesis of obesity and prediabetes has been extensively studied, in both *in vitro* models and in humans. It is to be noted that one miRNA can simultaneously regulate numerous target genes, leading to intricate regulatory networks in the development of prediabetes or obesity. For example, miR-192 is linked to insulin resistance, adipocyte differentiation, and inflammation in obese prediabetes individuals [41,45,49]. There are several common mechanisms that lead obese individuals to developing prediabetes and eventually T2DM. The transition from obesity to prediabetes involves interconnected processes including chronic low-grade inflammation, insulin resistance, β-cell stress, and ectopic lipid accumulation. Adipose expansion in obesity promotes dysregulated adipokine secretion and macrophage infiltration, leading to release of inflammatory mediators such as IL-6 and TNFα. These signals impair insulin signaling in liver, muscle, and adipose tissue, while simultaneously increasing β-cell workload to maintain normal blood glucose in the body. As a result, prolonged exposure to inflammatory and lipotoxic stress ultimately leads to β-cell dysfunction and impaired glucose tolerance. Within this mechanistic framework, several circulating microRNAs may be a potential biomarker of these underlying biological processes.

**Table 2:** Mechanistic mapping of priority miRNAs

miRNA	Primary pathway(s)	Direction in prediabetes/Obesity	Key targets/Nodes	Overlap with other conditions	Implication for specificity
miR27 [46,49]	Inflammation/oxidative stress	↑ in obese prediabetes	BCL2, BMI1, FOXO3, PTEN Lower SIRT1, increase IL-6	-	Adjusted for weight [49]
miR-34a [43,45]	Inflammation	↑ in obese prediabetes	TNFα, procalcitonin SFRP4	NAFLD	-
miR-126 [39,40,49]	β-cell function	↑ in obese, ↓ in prediabetes [39] ↓ in obese [40]	BCL2	Endothelial dysfunction	-
miR-146a [38,39,43]	β-cell function Inflammation	↑ in obese [39] ↓ in prediabetes [43]	IL-6, TNFα	-	-
miR-192 [41,45,49]	Insulin resistance, TGF‐β signaling Adipocyte differentiation Inflammation	↑ in obese prediabetes	PIK3R4, ACVR2B BCL2 TNFα, IL-1β, procalcitonin	NAFLD [45]	-
miR-30a [40,44]	β-cell dysfunction Cell death	↑ in prediabetes [44] ↑ in obese [40]	Beta2/NeuroD	-	-
miR-93 [39,47]	Insulin resistance	↑ in obese prediabetes [47] ↓ in obese [39]	GLUT4 IL-8	-	-
miR-122 [41,45]	Insulin signaling Inflammation	↑ in obese prediabetes	AKT3, IGF1R TNFα, IL-1β, procalcitonin	NAFLD [45]	Strong in liver-associated pathways
miR-152 [39,47]	Inflammation	↑ in obese [39] ↑ in obese prediabetes [47]	SFRP4	-	-

Obesity creates a pro-inflammatory adipose microenvironment, with macrophage infiltration and cytokine release. Several miRNAs mirrored this state. miR-27 was up-regulated in obese prediabetes, targeting regulators of oxidative stress and apoptosis such as BCL2, BMI1, FOXO3, and suppressing SIRT1, thereby amplifying IL-6 production [46,49]. miR-34a showed consistent elevation across obese prediabetes cohorts, targeting TNFα, procalcitonin, and Wnt signaling modulators such as SFRP4 [39,43,45]. miR-146a displayed context-specific changes, where it is increased in obesity, but reduced or mixed in prediabetes, suggesting stage-dependent modulation of inflammatory signaling through IL-6 and TNFα [38,39,43]. Collectively, these inflammation-linked miRNAs likely capture the transition from metabolically healthy obesity toward metabolically unhealthy obesity. However, their elevation is not unique to glycemic deterioration, since they are also up-regulated in conditions such as NAFLD [45] underscoring the risk of false positives in clinical diagnosis.

β-cell dysfunction is closely linked to dysglycemia and insulin resistance. miR-126 was consistently down-regulated in prediabetes but paradoxically elevated in obesity, suggesting dual roles in endothelial support and β-cell viability [39,40,49]. miR-30a was also increased, associated with β-cell dysfunction through Beta2/NeuroD regulation and pro-apoptotic pathways [40,44]. These miRNAs reflect early β-cell compensation that eventually fails under chronic metabolic stress, highlighting their potential as early indicators of impaired insulin secretion.

Furthermore, several miRNAs mapped directly to insulin signaling cascades. miR-192 was up-regulated in obese prediabetes and targeted PIK3R4, ACVR2B, and inflammatory mediators such as TNFα and IL-1β [41,45,49]. miR-93 has inconsistent results across papers, where it is up-regulated in obese prediabetes [47] but down-regulated in obese normoglycemia [39]. miR-93 targets GLUT4 and inflammatory chemokines such as IL-8 [39,47]. miR-122 was consistently elevated and strongly linked to hepatic insulin resistance, regulating AKT3 and IGF1R. Its overlap with NAFLD underscores that liver-specific signals may lack specificity for dysglycemia [39,41,45].

The overlap of these miRNAs across obesity and prediabetes demonstrates both their mechanistic plausibility and diagnostic challenge. Obesity itself drives widespread miRNA dysregulation, and single-miRNA signatures may not distinguish prediabetes from other inflammatory states. Therefore, emerging strategies to improve specificity are needed. For future research, it is suggested to use composite multi-miRNA panels, which capture multiple mechanistic axes simultaneously, enhancing specificity. Besides, integration with clinical/biochemical covariates such as HOMA-IR, waist-to-height ratio, or inflammatory markers. Validation against disease controls such as NAFLD, PCOS, and systemic inflammation rather than only against healthy comparators can help to narrow down specific miRNAs that are dysregulated among obese prediabetes individuals. Combining these three aspects, potential miRNAs for prediabetes among the high-risk individuals could be identified as biomarkers for clinical diagnosis or risk stratification.

### Techniques for microRNA detection and quantification

#### qRT-PCR

Quantitative real-time polymerase chain reaction (qRT-PCR)-based methods are useful for the detection and quantification of miRNAs, yielding high efficiency, sensitivity, and specificity. Furthermore, qRT-PCR has a large dynamic range and can differentiate RNA expression between samples [50]. It is considered the gold standard approach in identifying circulating miRNAs. The principle of qRT-PCR focuses on two steps: synthesis of cDNA using reverse transcriptase followed by conventional real-time PCR to detect amplified products using SYBR Green fluorescent dye or TaqMan probe [51]. The most common method to detect mature miRNA is using stem-loop primer during cDNA synthesis as it can differentiate miRNA species. First, the stem-loop primer is annealed to the 3′ end of miRNA to be reverse transcribed. After RNA degradation of the original miRNA, the transcribed sequence will be used as a template for miRNA amplification. Then, qPCR will be carried out using SYBR Green dye or Taqman probe to monitor the fluorescent signal. SYBR Green is an intercalating dye that enhances its fluorescence signal up to 1000 times when bound to double-stranded DNA, whereas the Taqman probe is a probe that is complementary to the DNA and comes with a fluorophore at the 5′ end and a quencher at the 3′ end. In this case, the Taqman probe works by binding to the target DNA strand between the forward and reverse primer, so that during amplification, the probe will be displaced, and fluorophore will be released, which increases the fluorescence signal. Comparing SYBR Green dye with the Taqman probe, the Taqman probe is more accurate and specific as it will not amplify nonspecific products such as primer dimers [52]. Overall, qRT-PCR is highly sensitive and useful in quantifying targeted miRNAs. However, the challenge lies in difficult primer design and inconsistent data analysis, which affects the reproducibility of qRT-PCR [50].

#### Microarrays

Microarrays are a well-known high-throughput method for miRNA expression profile comparison. The strengths of microarrays are rapid and able to quantify and assess the expression of hundreds of miRNAs simultaneously in a single assay [51]. The general principle of microarray is the binding of complementary sequences known as hybridization [53]. The miRNA microarray is prepared by immobilizing miRNA oligonucleotide probes onto glass slides. Then, the isolation of miRNAs is done by reverse transcription with simultaneous labeling with a fluorophore. When the labeled miRNAs are added to the prepared glass slides, hybridization between miRNAs and probes occurs, which results in fluorescence emission at different spots on the slides. Therefore, the expression level of miRNAs in the sample can be studied by analyzing the fluorescence signal intensity [54]. In this context, microarrays are useful in measuring the differential expression of miRNAs to determine the pathophysiological status of cellular microenvironment and compare miRNA expression in different organs and tissues [51]. However, the limitations of using microarray to detect miRNA are high cost and relatively low sensitivity. Other challenges include low specificity when analyzing miRNAs with similar sequences, and the detection of short miRNAs is poor [52].

#### Next-generation sequencing

Sequence-based methods are extremely sensitive in the detection of small molecules with short sequence reads. Next-generation sequencing (NGS) can provide an expression profile of known miRNAs, as well as identify unknown miRNA sequences [50]. It starts with extracting miRNA fragments from the total RNA obtained from the sample, followed by size selection to exclude all bigger fragments. Then, ligation of sequencing adapters will be done, followed by reverse transcription into cDNA. The output of NGS contains millions of short reads that need to be processed and interpreted using different software. In short, the data processing steps include aligning to a reference genome, removing the adapter, filtering out low-quality reads and other noncoding small RNA species, and lastly computing miRNA expression levels based on the read counts in each sample. Then, miRNA expression analysis can be performed by using tools like miRExpress, MirTools, and deepBase. Moreover, using the sequence of mature miRNAs, prediction of miRNA target can be done by comparing it with sequences of mRNA candidate target. The principle involves complementary binding of nucleotides, where high complementary binding will lead to target degradation and mismatches in the binding will lead to translational repression. The abundance of reads between miRNA and the target mRNA is expected to be inversely correlated as the target mRNA is usually degraded upon the binding of miRNA, which indicates high complementary binding [55]. The main challenge that limits the use of NGS in the detection of miRNA is cost. It is too expensive for routine clinical use and requires computational infrastructure to analyze and interpret the data generated [50].

## Discussion

An ideal biomarker should be easily accessible through minimally invasive procedures, have high specificity and sensitivity, and be translatable from research to clinical settings [56]; miRNAs meet many of these criteria. They demonstrate high stability in biological fluids such as plasma, making them promising candidates for diagnostic biomarkers in metabolic diseases. In addition, miRNAs are resistant to degradation and can withstand multiple freeze-thaw cycles and extreme pH levels, which enhances their practicality in clinical use [57]. miRNAs also exhibit high tissue or cell-type specificity, with certain miRNAs, such as miR-133b and miR-1–3p in muscle, miR-122–5p in the liver, and miR-375 in pancreatic β-cells, demonstrating clear associations with specific tissues [58,59]. For instance, miR-1 and miR-133 show high sensitivity (96.4%) and specificity (81.5%) in distinguishing prediabetes from healthy controls [60]. Although HbA1c levels are well-established diagnostic markers for prediabetes, misdiagnoses often occur in the early stages since these markers typically become detectable only as the disease progresses. HbA1c diagnostic performance varies across populations, with a general range between 0.61 and 0.74 [28,61].

Previous reviews, such as González-Sánchez et al. [62], primarily emphasized the broad involvement of circulating miRNAs in type 2 diabetes pathophysiology, with less focus on the prediabetic stage and the influence of obesity [62]. Similarly, Zhu and Leung (2023) provided a comprehensive overview of circulating miRNAs as emerging metabolic biomarkers, but their synthesis largely concentrated on diabetes diagnosis and complications rather than early dysglycemia [63]. In contrast, our review specifically targets prediabetes in the context of obesity, highlighting candidate miRNAs such as miR-34a, miR-146a, and miR-30a, and integrating mechanistic roles with diagnostic performance metrics across diverse populations. Our synthesis indicates that circulating miRNAs hold mechanistic and diagnostic relevance for obesity-related prediabetes, but their translation remains constrained by methodological and demographic heterogeneity. Quantitatively, single miRNAs demonstrated modest accuracy (AUC 0.60–0.75), whereas multi-miRNA panels reached stronger discrimination (AUC up to 0.81), particularly in obese subgroups.

However, demographic generalizability is limited. Several cohorts were female-only or geographically narrow, and very few studies explored sex or ethnicity as effect modifiers. Furthermore, there is methodological variability, including inconsistent preanalytics, normalization strategies, and thresholds, which further reduces reproducibility. Only a few datasets reported calibration or decision-curve analyses, and external validation was largely absent. Despite promising mechanistic and diagnostic insights, circulating miRNAs remain at an early stage of clinical translation for prediabetes. A major challenge is the lack of standardization. Preanalytical factors such as fasting status, hemolysis control, and sample storage, as well as extraction and normalization methods, vary across studies. Therefore, standardized protocols and MIQE-compliant reporting in future studies will be essential to ensure reproducibility and comparability.

Future research should also focus on including diverse demographic groups to evaluate the generalizability of miRNA biomarkers across different populations [64]. Additionally, the influence of environmental and lifestyle factors on miRNA expression in the context of obesity and prediabetes warrants further investigation [65]. Moreover, longitudinal studies could track changes in miRNA profiles over time in individuals at risk of developing prediabetes, offering insights into disease progression [66]. It is also worthwhile to explore the combination of multiple miRNAs [67], as well as integrating miRNA biomarkers with other biological markers or current diagnostic methods, to potentially enhance the accuracy of early detection [68].

For clinical workflow, miRNA testing could serve several roles. For instance, miRNA profiling may be most useful as an adjunct to HbA1c or fasting glucose, potentially guiding decisions on oral glucose tolerance testing or refining obesity-specific risk stratification. Yet, no study has directly established incremental value over conventional markers. Nonetheless, regulatory translation will require a stepwise path of analytical validation, clinical validation, and demonstration of clinical utility within frameworks such as CLIA and in vitro diagnostic (IVD) approval pathways. From an assay perspective, qRT-PCR remains the most commonly used platform due to accessibility and cost-effectiveness, but droplet digital PCR (ddPCR) and targeted next-generation sequencing (NGS) may offer improved sensitivity, dynamic range, and multiplexing capacity, but it also comes with higher per-sample costs and longer turnaround times. Another major research gap that must be addressed before widespread adoption is that health economic modeling is needed to assess whether miRNA-based strategies provide cost-effective improvements over current approaches.

## Conclusion

Emerging evidence supports circulating miRNAs as promising biomarkers for the early detection of prediabetes, particularly within obese populations where traditional glycemic markers may lack sensitivity. Mechanistic studies highlight their involvement in insulin resistance, β-cell dysfunction, and adipose tissue inflammation, providing a strong biological background. However, translational readiness remains limited by preanalytical variability, lack of assay standardization, and limited external validation. By integrating mechanistic insights with practical considerations of assay performance, regulatory approval, and cost-effectiveness, future research can provide higher chances for circulating miRNAs to progress from exploratory markers to clinically applicable tools in prediabetes risk stratification. Looking at the limitations of past research, combining miRNA signatures with multiomics profiling, machine learning–based risk prediction, and longitudinal cohort validation may accelerate their integration into personalized medicine and population-level screening strategies.

## Abbreviations

DM: diabetes mellitus
OGTT: oral glucose tolerance test
T2DM: type 2 diabetes mellitus
miRNAs: microRNAs
